# Luminescence and stability of Tb doped CaF_2_ nanoparticles†

**DOI:** 10.1039/d2ra07897j

**Published:** 2023-02-13

**Authors:** E. H. H. Hasabeldaim, H. C. Swart, R. E. Kroon

**Affiliations:** a Department of Physics, University of the Free State PO Box 339 Bloemfontein 9300 South Africa omda180@gmail.com KroonRE@ufs.ac.za

## Abstract

Luminescence properties of CaF_2_:Tb^3+^ nanoparticles were studied in order to investigate the effect of CaF_2_ native defects on the photoluminescence dynamics of Tb^3+^ ions. Incorporation of Tb ions into the CaF_2_ host was confirmed by X-ray diffraction and X-ray photoelectron spectroscopy. Cross-relaxation energy transfer was observed from the photoluminescence spectra and decay curves upon excitation at 257 nm. However, the unusual long lifetime of the Tb^3+^ ion as well as the decreasing trend of emission lifetime of the ^5^D_3_ level suggested the involvement of traps, which were further investigated by using temperature-dependent photoluminescence measurements, thermoluminescence and lifetime measurements at different wavelengths. This work highlights the critical role that the CaF_2_ native defects play in the photoluminescence dynamics of Tb^3+^ ions incorporated in a CaF_2_ matrix. The sample doped with 10 mol% of Tb^3+^ ions was found to be stable under prolonged 254 nm ultraviolet irradiation.

## Abstract 2

Luminescence properties of CaF_2_:Tb^3+^ nanoparticles were studied in order to investigate the effect of CaF_2_ native defects on the photoluminescence dynamics of Tb^3+^ ions.

## Graphical abstract

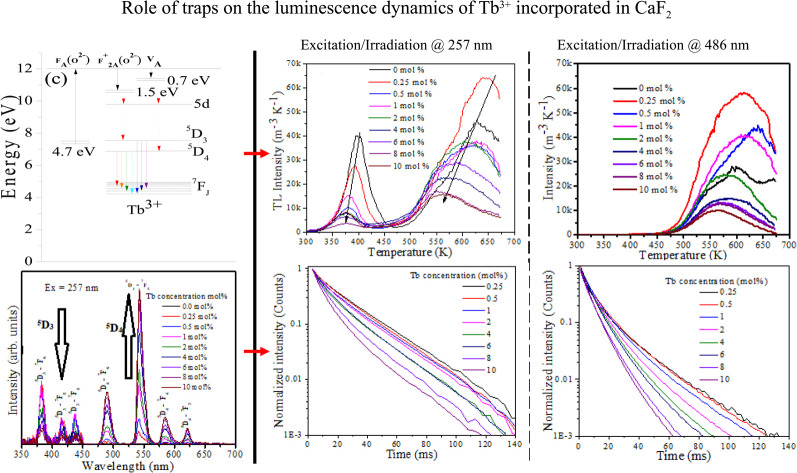

## 1 Introduction

Lanthanide (Ln) doped phosphors are an important class of luminescent materials because of their compatibility for use in numerous applications.^1^ The sharp 4f–4f band emission of Ln^3+^ ions and their long-lived photoluminescence (PL),^2^ low toxicity, and stability against photobleaching make them suitable for use in lighting, photovoltaic, laser, and bio-imaging applications.^3–5^ Fluoride host materials, owing to their intrinsic properties including low phonon energy (<400 cm^−1^), low refractive index, high transparency,^6^ and wide band gap are considered suitable hosts for Ln^3+^ ions to obtain efficient PL. CaF_2_ has a wide optical bandgap (12.1 eV) and a well-known face centred cubic crystal structure (where each Ca^2+^ is coordinated with eight F^−^ anions, and each F^−^ is coordinated by four Ca^2+^ cations) and is considered to be a model host for Ln^3+^ ions.^7^

Tb^3+^ is a well-known activator ion, owing to its blue and green emissions originating from the ^5^D_3_–^7^F_*J*_ (*J* = 6, 5, 4) and ^5^D_4_–^7^F_*J*_ (*J* = 6, 5, 4, 3) multiplet transitions, respectively. Its stimulated emission at 544 nm renders it a suitable activator for green solid state laser materials.^8^ Tuning the green to blue emission ratio of Tb^3+^ ions is achieved *via* cross-relaxation energy transfer from the ^5^D_3_ to ^5^D_4_ level.^9^ This mechanism of energy transfer depends on the interaction between Tb^3+^ ions which occurs when the distances between the Tb^3+^ ions are sufficient short, and it is a concentration dependent phenomenon.^10^ The efficient luminescence from Tb^3+^ ions in the CaF_2_ host is hindered by two reasons: (i) due to the charge difference between Tb^3+^ ions and Ca^2+^ ions, an effective substitution is limited to low concentrations, and hence increasing the doping concentration may result in the formation of undesirable complex defects, (ii) the native point defects present in CaF_2_ crystals act as electron traps, and they interact with foreign ions and lead to the formation of other complex defect structures.^11^ These phenomena introduce more nonradiative paths which reduce the emission intensity of the Tb^3+^ ions.

Native point defects of CaF_2_ have been investigated for a very long time. Numerous theoretical and experimental approaches have been devoted to elucidating the nature of such defects. The fluorine centre is found to be one of the most prominent defects and it form complexes when it is perturbed by the parasitic oxygen ions that are always present in CaF_2_ crystals.^12–15^ On the other hand, recent research is focused on doping CaF_2_ with different rare earth ions in order to develop an excellent CaF_2_-based photonic material for various applications such as lasers, bioimaging, lighting, thermoluminescence, *etc.* Nevertheless, a systematic investigation addressing the role of these defects on the luminescence dynamics of rare earth ions incorporated in CaF_2_ host has rarely been performed. Zheng *et al.* reported an unusual long lifetime in the order of milliseconds for CaF_2_:Ce^3+^, Tb^3+^ nanocrystals with different Na^+^ concentrations.^3^

In this work, luminescence properties of Tb^3+^ doped CaF_2_ nanoparticles were studied. The effects the CaF_2_ point defect and Tb concentration on the luminescence dynamics and cross-relaxation energy transfer of the Tb^3+^ ions were discussed. The stability of Tb^3+^ doped CaF_2_ under UV irradiation was also investigated.

## 2 Experimental

### 2.1 Materials and synthesis

The CaF_2_:Tb^3+^ nanoparticles were synthesized by using the hydrothermal method. The starting materials included calcium nitrate tetrahydrate (Ca(NO_3_)_2_·4H_2_O), ammonium fluoride (NH_4_F) and terbium nitrate pentahydrate (Tb(NO_3_)_3_·5H_2_O), which were purchased from Sigma Aldrich and used without further purification. For synthesis 10 mmol of calcium nitrate and 20 mmol of ammonium fluoride were separately dissolved in 70 ml and 50 ml of deionized water, respectively, and kept under magnetic stirring for 30 min. The ammonium fluoride solution was added dropwise to the calcium nitrate solution, and the resulting solution was further stirred for another 20 min until it became cloudy. This solution was transferred into a Teflon container which was put into a Yanzheng instrument microreactor and thoroughly sealed. The reaction was performed at 160 °C for 5 h under continuous magnetic stirring, after which it was cooled to room temperature. The CaF_2_ nanoparticles were isolated using a centrifuge at 6000 rpm for 10 min, and washed with ethanol and water four times. The final product was dried in an oven at 70 °C for 24 h. For the synthesis of Tb doped CaF_2_, different amounts of terbium nitrate were added relative to the calcium nitrate, and the synthesis procedures were maintained the same as the standard sample.

### 2.2 Characterization

A Bruker D8 instrument was used for the X-ray diffraction measurements. Chemical analyses were performed with X-ray photoelectron spectroscopy (XPS) by using a PHI 5000 Versaprobe-Scanning ESCA Microprobe. A 100 μm diameter monochromatic Al Kα X-ray beam (1486.6 eV) was used for the measurements. For the wide survey scans and high-resolution spectra, the hemispherical analyzer pass energy was maintained at 187 eV and 11.8 eV respectively for 3 cycles. The measurements were performed using 1 eV per step and 0.1 eV per step for wide survey scans and high-resolution scans, respectively. The PL data (excitation, emission, and decay curves) were recorded by using a Cary Eclipse fluorescence spectrophotometer with a xenon lamp as the excitation source in the phosphorescence mode. A 325 nm He–Cd laser was utilized to record PL spectra at different temperatures. A Philips CM100 Analytical Transmission Electron Microscope (TEM) was used to obtain information about the size distribution of the nanoparticles. Thermoluminescence (TL) glow curves were measured by using a Nucleonix system with heating rate of 5 K s^−1^. A 254 nm UV lamp and USB200 Ocean Optics spectrometer were utilized for the prolonged UV irradiation experiment.

## 3 Results and discussions

### 3.1 X-ray diffraction analysis

**Fig. 1:**
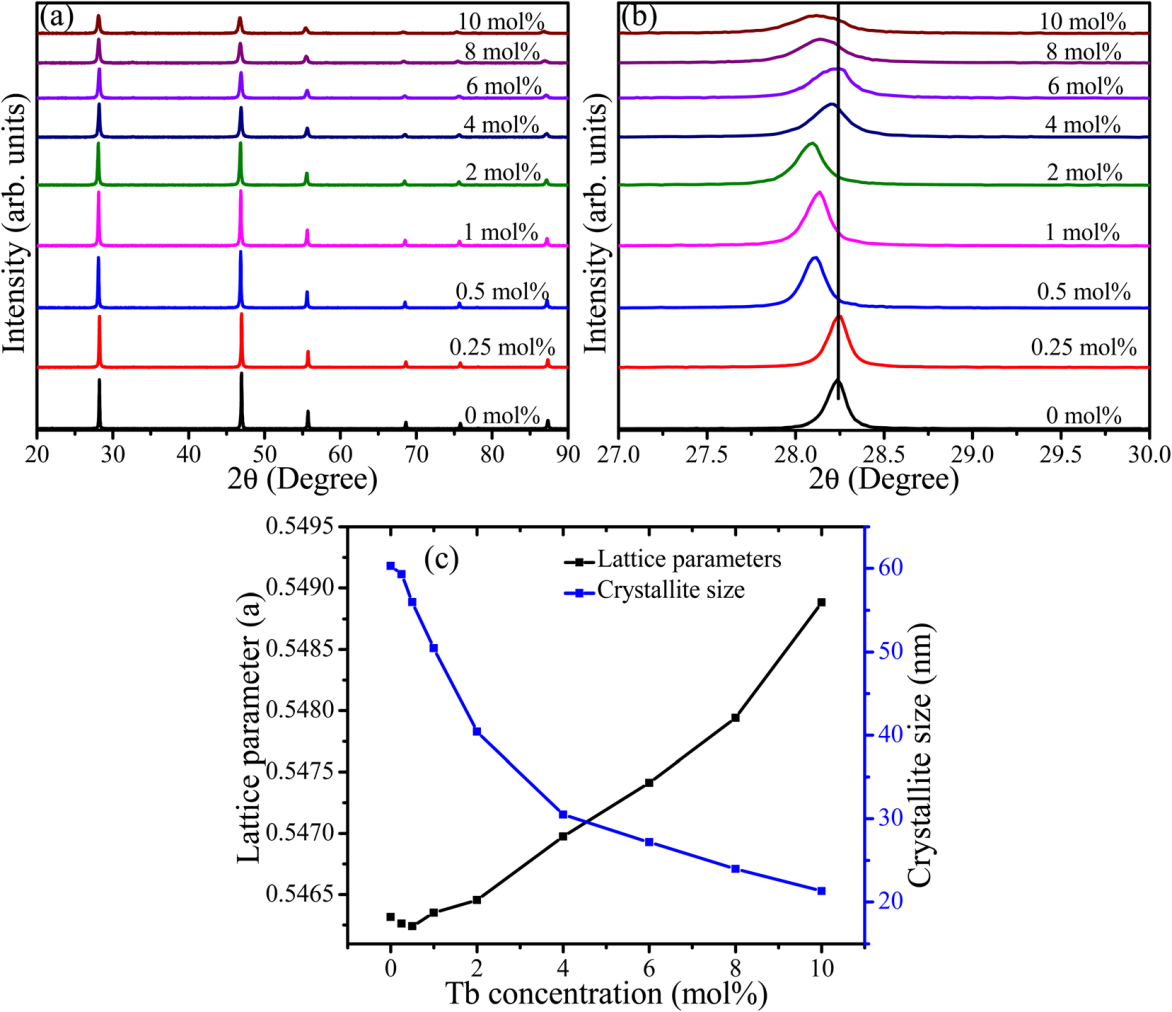
(a) XRD patterns of CaF_2_ nanoparticles doped with different concentrations of Tb^3+^ ions, (b) zoomed-in region of the 111 peak, and (c) the calculated lattice parameters and crystallite sizes.

XRD patterns of CaF_2_ doped with different Tb concentrations are depicted in Fig. 1(a). The patterns of all samples showed reflections near 2*θ* angles of 28.2°, 47.0°, 55.7°, 68.7°, 75.9°, and 87.3°, which according to JCPDS 00-004-0864 correspond to the (111), (220), (311), (400), (331), and (422) planes of cubic CaF_2_, respectively. No extra peaks associated with other phases were observed, which indicates the substitution of Ca^2+^ ions by Tb^3+^ ions. The overall peak intensities showed a decreasing trend upon increasing the Tb concentration, which indicates deterioration of crystallinity and reduction in the crystallite size. For further investigation, Rietveld refinement was performed for all samples and the fittings provided in the ESI (Fig. S1†). The lattice parameter initially decreased very slightly at low doping concentrations (0.25 and 0.5 mol%) and then increased as the doping concentration increased further (Fig. 1(c)). The initial decrease of the lattice constant can be ascribed to the incorporation of Tb^3+^ ions into CaF_2_ lattice *via* substitution. Substitution of Ca^2+^ ions (ionic radius 99 pm) with smaller ions such as Tb^3+^ (92 pm) would generally be expected to induce compressive stress in the crystal lattice and, as a result, the lattice constant shrinks. The expansion of lattice parameters at higher Tb concentration indicates that another mechanism may have been involved in the substitution process. Ionic substitution accompanied by creation of interstitials for charge compensation could be the other mechanism responsible for the expansion of the lattice parameter. When a cation (Ca^2+^) with lower charge substituted by another smaller cation (Tb^3+^) with larger charge, an interstitial anion defect (F^−^ with ionic radius 136 pm) is created in order to maintain charge neutrality according to the substitutional mechanism: Ca^2+^ → Tb^3+^ + F^−^.^16^ The inconsistency of the 111-peak position (Fig. 1(b)) is due to the instrumental contribution. Scherrer's equation was used to estimate the crystallite sizes of the samples with different Tb concentrations and it was found that the crystallite size decreased from about 60 to 20 nm as the Tb concentration increased from zero to 10 mol% (Fig. 1(c)). This is attributed to the incorporation of foreign ions with different ionic radii and creation of interstitial fluorine centres with larger ionic radii which perturb the crystal and inhibit further crystal growth.

### 3.2 Surface morphology

**Fig. 2:**
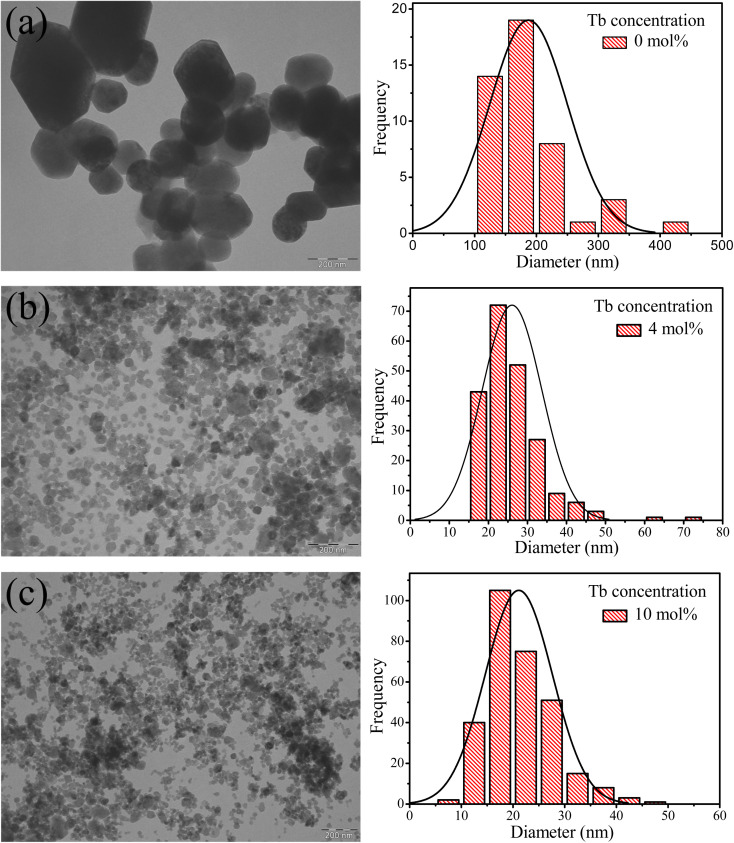
TEM images of CaF_2_ nanoparticles doped with different Tb^3+^ contents (a) 0 mol%, (b) 4 mol% and (c) 10 mol%. Corresponding histograms represent the particles size distributions obtained from the TEM images.

Fig. 2 displays TEM images of samples with zero, 4 and 10 mol% Tb. The undoped CaF_2_ sample exhibited an irregular particle shape with an average particle diameter of about 188 nm, substantially greater than the crystallite size which indicates that each particle was composed of many grains. The average particle size decreased to 26 nm and 21 nm for the 4 and 10 mol% doped samples, respectively, meaning that in the highly doped sample the particle size and crystallite size are comparable. Substitution of Ca^2+^ ions by Tb^3+^ ions *via* the charge compensation mechanism is the major reason for the reduction of the crystallinity and particle size. When an ion with different ionic radius is introduced into a lattice or an interstitial defect is created, the lattice geometry is distorted and hence further crystal and particle growth is inhibited.

### 3.3 X-ray photoelectron spectroscopy

**Fig. 3:**
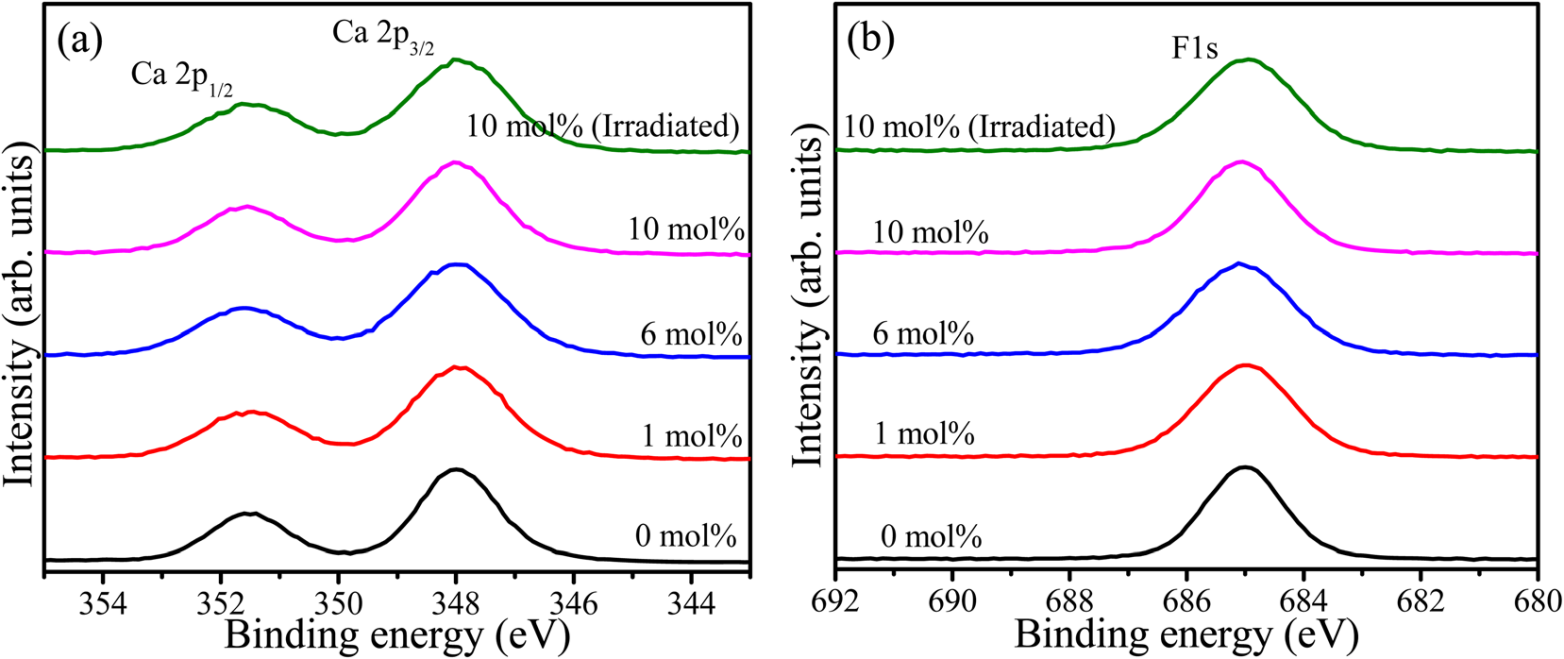
XPS high resolution spectra of (a) Ca 2p and (b) F 1s of the CaF_2_ doped with different Tb concentrations.

Wide survey XPS scan was recorded for samples containing 0, 1, 6, and 10 mol% of Tb as shown in the ESI (Fig. S2†). The principal elements, including Ca, F, and Tb, were detected as well as carbon due to atmospheric hydrocarbon contamination. The oxygen peak was also detected and can be attributed to the parasitic oxygen of CaF_2_ and surface contaminations. The relative concentration of the detected elements was calculated^17^ and tabulated in Table 1. These values may deviate from the bulk chemical composition because XPS is sensitive to the overlaying surface contaminations layer. High resolution XPS spectra of Ca 2p for the samples with different Tb concentrations as well as the UV-irradiated sample are shown in Fig. 3(a). These exhibited the spin–orbit doublet due to Ca 2p_3/2_ and Ca 2p_1/2_ with binding energies 348.0 eV and 351.6 eV, respectively.^18^ This suggests the presence of calcium ions in the divalent oxidation state (Ca^2+^).^19^ The F 1s high resolution XPS spectra of the same samples are shown in Fig. 3(b). The symmetrical feature of the F 1s peaks signify that only one component of F associated with Ca–F is present in the material. The full width at half maxima (FWHM) of the XPS peaks associated with Ca 2p and F 1s, given in Table 1, were increased for the samples containing Tb ions and was also slightly increased after UV irradiation. This may be facilitated by factors such as changes in the chemical environment and creation or annihilation of defects as a result of Tb addition and UV irradiation.

**Table 1:** Widths (FWHM) of XPS peaks and atomic ratio of the principal elements

Sample	Ca 2p_3/2_ (eV)	F 1s (eV)	Atomic ratio (%)			
			Ca	F	O	Tb
CaF_2_	1.9	1.7	31.5	62.3	6.2	
CaF_2_:Tb 1 mol%	2.3	2.1	31.2	62.1	6.3	0.4
CaF_2_:Tb 6 mol%	2.4	2.2	29.1	61.6	5.2	4.1
CaF_2_:Tb 10 mol%	2.2	2.0	27.8	61.0	4.6	6.6
CaF_2_:Tb 10 mol% (irradiated)	2.3	2.2	27.9	60.6	5.0	6.5

**Fig. 4:**
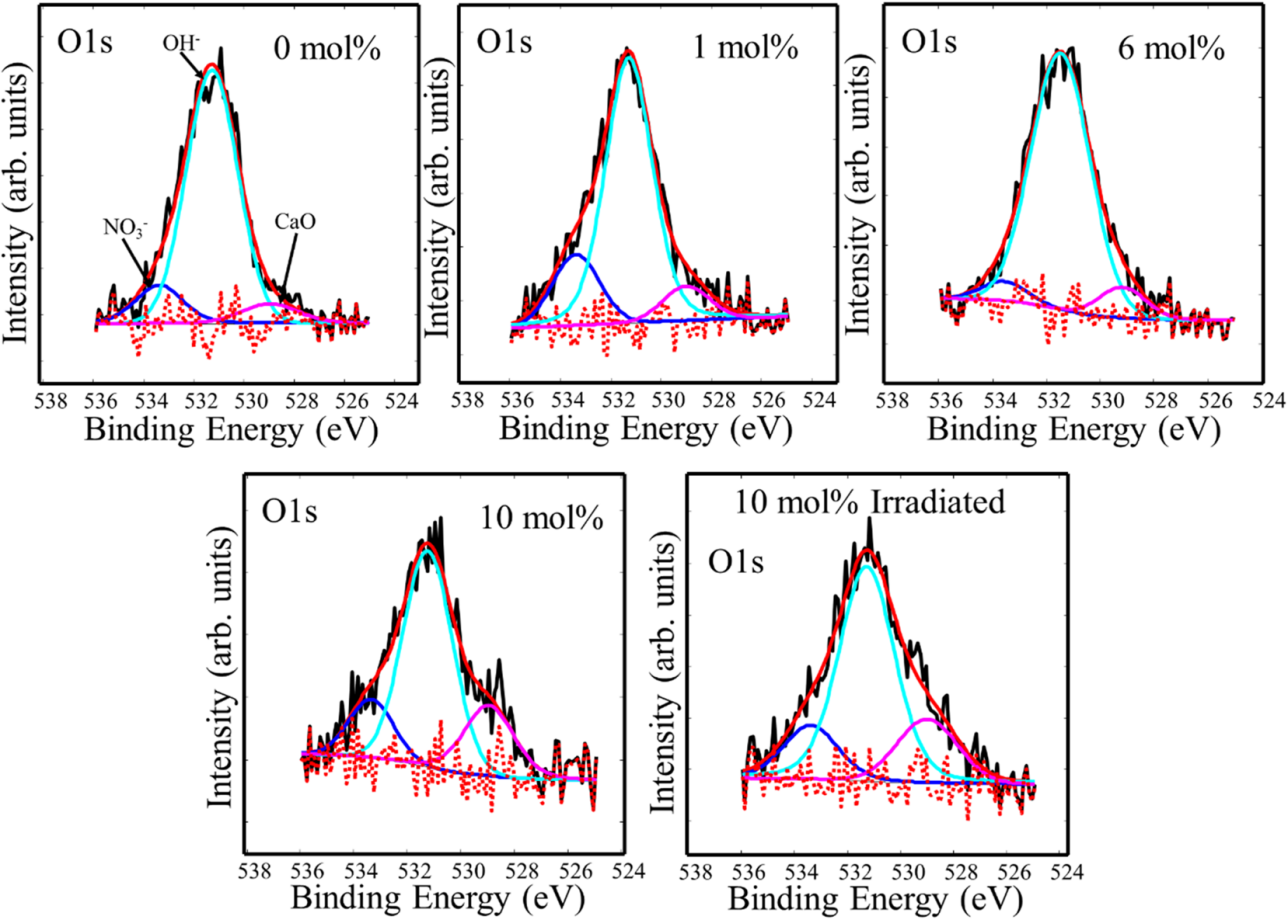
XPS high resolution spectra of O 1s impurity in CaF_2_ doped with different Tb concentrations.

Fluoride compounds synthesized by the hydrothermal method are often contaminated with a small amount of oxygen and XPS high resolution spectra of the O 1s binding energy region are presented in Fig. 4. Three component peaks were deconvoluted at 533.4 eV, 531.3 eV, and 529.0 eV which were attributed to ionic NO_3_^−^, OH^−^, and oxygen bonded to metal (CaO), respectively.^13^ This indicates the presence of oxygen with different forms, but mainly hydroxide, in the CaF_2_ matrix which may also add to the native defects' complexity. Since XRD measurements did not detect any phase associated with these oxygen-related materials, their detection by XPS could indicate their presence primarily on the surface. OH^−^ can easily be cascaded into F^−^ sites due to their similar ionic radius of 1.35 Å and 131 Å for OH^−^ and F^−^, respectively.^20^ Peaks positions and relative areas of the O 1s components are listed in Table 2.

**Table 2:** Energies and relative areas of O 1s and Tb 3d deconvolution components

Peak type	O 1s			Tb 3d	
Sample	533.4 eV	531.3 eV	529.0 eV	1240.5 eV	1242.8 eV
CaF_2_	12	80	8	—	—
CaF_2_:Tb 1 mol%	18	74	8	53	47
CaF_2_:Tb 6 mol%	5	87	8	57	43
CaF_2_:Tb 10 mol%	15	63	22	62	38
CaF_2_:Tb 10 mol% (irradiated)	16	65	19	62	38

**Fig. 5:**
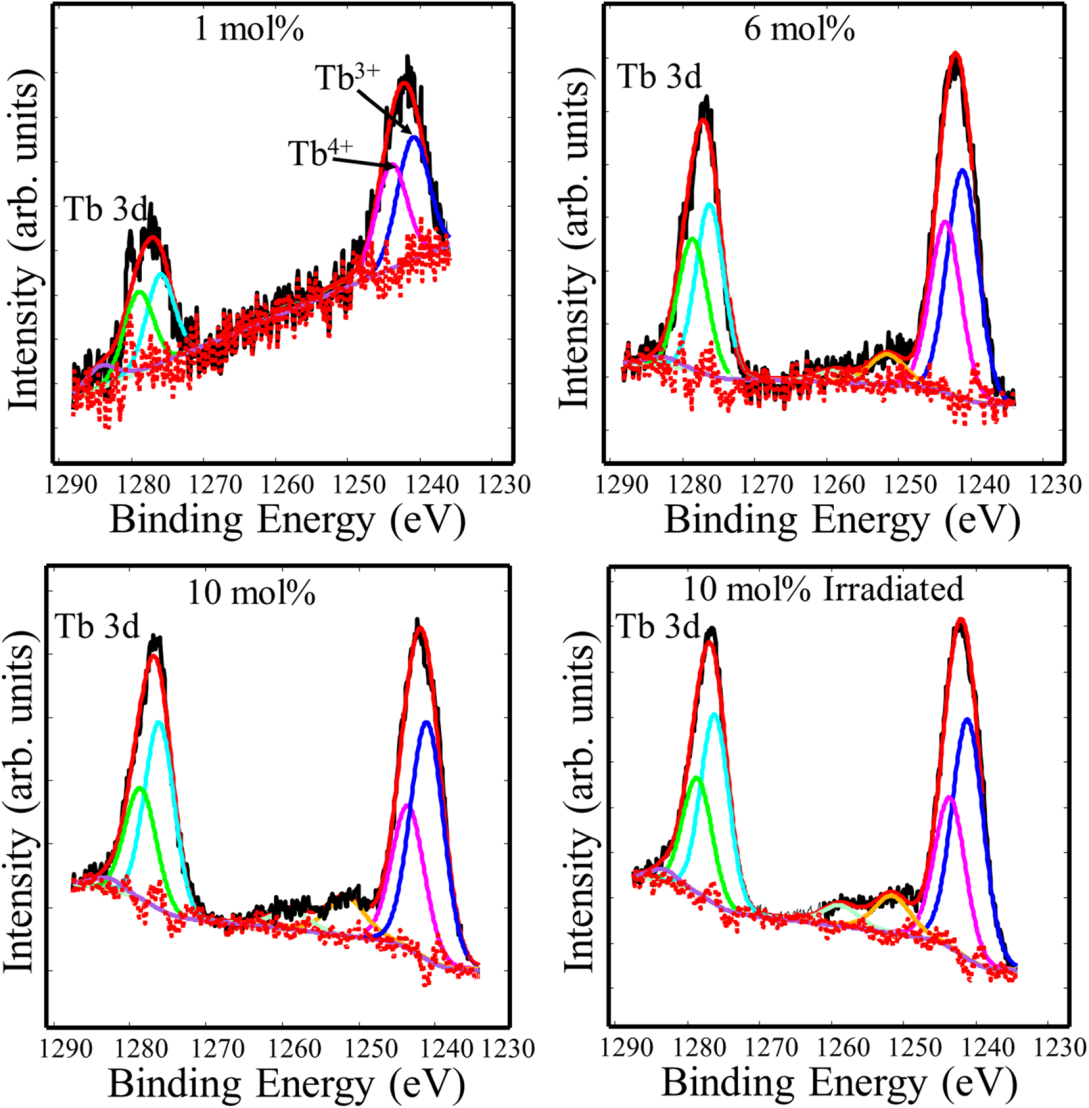
XPS high resolution spectra of Tb 3d of the CaF_2_ doped with different Tb concentrations.

In order to study the valence state of Tb ions in CaF_2_, high resolution spectra of the Tb 3d binding energy region were recorded. The Tb^3+^ 3d main peak split into two major peaks due to the spin–orbit interaction, whose binding energies were 1240.5 eV and 1275.5 eV.^21^ According to Gaussian–Lorenz fits, another two peaks at 1242.8 eV and 1277.8 eV were found and ascribed to tetravalent Tb^4+^.^22^ The small peaks at the higher binding energy sides relative to the major peaks may be credited to satellite phenomena.^23^ To understand the effect of doping concentration and UV irradiation, quantitative analysis was performed and the relative areas of the Tb^3+^ and Tb^4+^ deconvolution components were listed in Table 2. The relative peak area associated with trivalent Tb ions increased as the Tb doping increased, whereas it remained unchanged following UV irradiation. This indicates that Tb ions favoured the trivalent state at high Tb concentration, and the UV irradiation did not cause noticeable chemical changes (Fig. 5).

### 3.4 Photoluminescence

**Fig. 6:**
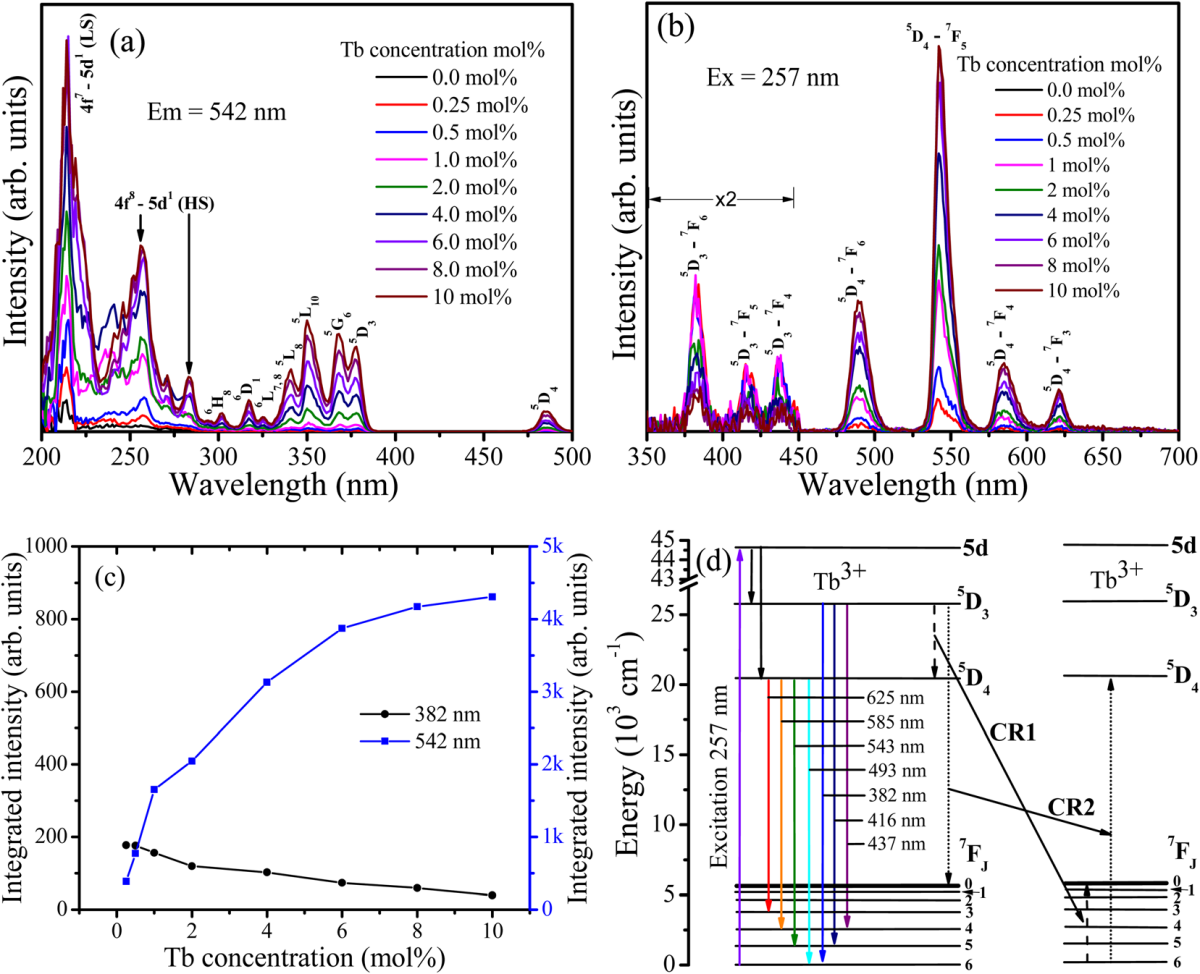
PL spectra of undoped and Tb^3+^ doped CaF_2_ nanoparticles. (a) Excitation spectra monitored for emission at 542 nm. (b) Emission spectra excited at 257 nm. (c) Integrated intensity of the bands at 382 nm and 542 nm as a function of Tb^3+^ concentration. (d) Energy diagram representation of Tb^3+^ ions in CaF_2_ host as well as the cross-relaxation paths.

Fig. 6 depicts the PL spectra of CaF_2_ doped with different Tb concentrations. The excitation spectra (Fig. 6(a)) consist of multiple bands. The bands in the range between 200 to 295 nm originate from the parity allowed 4f^8^–4f^7^ 5d^1^ transitions, whereas the other bands are attributed to the intra 4f–4f transitions of Tb^3+^ ions as marked in Fig. 6(a).^24^ The bands centred near 213 and 282 nm were ascribed respectively to the low spin (LS) and high spin (HS) exchange interaction between 5d electrons spin and 4f^*n*−1^ electrons total spin^25^. Upon excitation at 257 nm, the samples emitted two groups of emissions, blue emission in the region from 350 nm to 450 nm (*i.e.* at 382 nm, 416 nm, and 437 nm) which are ascribed to ^5^D_3_–^7^F_*J*_ (*J* = 6, 5, 4) transitions, and green emission in the region from 450 nm to 650 nm (*i.e.* at 490 nm, 542 nm, 585 nm, and 621 nm) which are attributed to ^5^D_4_–^7^F_*J*_ (*J* = 6, 5, 4, 3) transitions. The variation in the integrated intensity of the bands at 382 nm and 542 nm as a function of the Tb concentration is depicted in Fig. 6(c). The 542 nm band intensity increases as the Tb concentration increases, while the 382 nm intensity decreases. This indicates that cross-relaxation energy transfer from the ^5^D_3_ to ^5^D_4_ energy level takes place (Fig. 6(d)).

For the lower Tb concentrations of 0.25 mol% and 0.5 mol%, the 382 nm emission band intensity did not change, which means that the critical Tb^3+^ concentration at which cross-relaxation is effective is above 0.5 mol%. Increasing the Tb^3+^ concentration reduces the distance between Tb^3+^ ions and therefore the interactions between Tb^3+^ ions become stronger. This leads to an enhanced probability of cross-relaxation energy transfer. The energy difference between the ^5^D_3_ and ^5^D_4_ levels is about 5800 cm^−1^, which is much larger than 400 cm^−1^ phonon energy of fluoride compounds, signifying that phonon-assisted non-radiative relaxation between the levels is very unlikely and that cross-relaxation is the dominant relaxation process. Energy transfer interaction mechanisms between luminescent centres are generally classified into two categories: exchange interaction and electric multipole interactions. When the distance between luminescent ions is small enough (4 Å), exchange interaction dominates the energy transfer. Otherwise, electric multipole interactions prevail.^26,27^ The distance between the Tb^3+^ ions in CaF_2_ host can be estimated using 
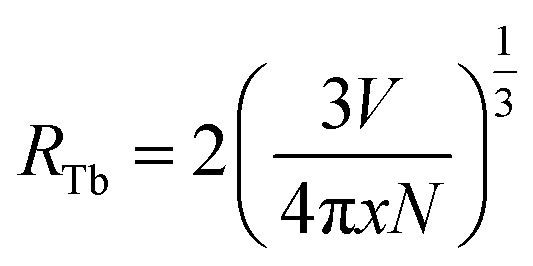
 where *V* represents the unit cell volume of CaF_2_ (163.4 Å^3^), *N* is the number of cationic sites that can be substituted by activators in one unit cell (which is 4 sites for CaF_2_) and *x* is the Tb activator concentration. The separation of Tb^3+^ ions is calculated to vary from 31.5 Å for the lowest Tb concentrations of 0.25 mol% to 9.2 Å for the highest Tb^3+^ ion concentration considered (10 mol%). Hence cross-relation occurs *via* the electric multipole mechanism rather than the exchange mechanism. CIE colour coordinates of the CaF_2_ samples containing different Tb concentrations were calculated and shown in the ESI (Fig. S3 and Table S1†).

**Fig. 7:**
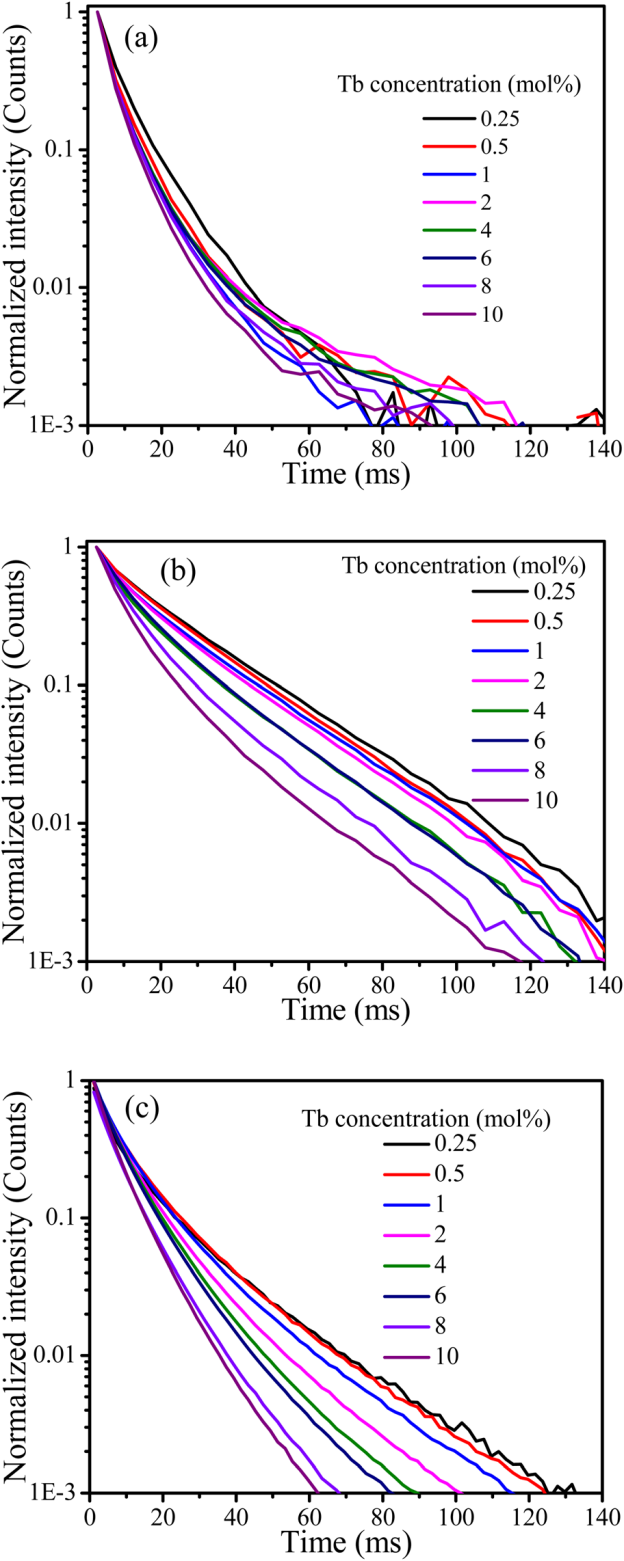
Decay curves of the CaF_2_ doped with different concentration of Tb ions excited at 257 nm for the emission band at (a) 382 nm and (b) 542 nm, (c) excited at 486 nm for the band at 542 nm.

Cross-relaxation between Tb^3+^ ions decreases the lifetime of the ^5^D_3_ emissions. To further analyse the cross-relaxation process, the decay curves of the ^5^D_3_ emission at 382 nm and the ^5^D_4_ emission at 542 nm were measured for the samples with different Tb ions concentration and are shown in Fig. 7. The average lifetimes were calculated using:^28^1
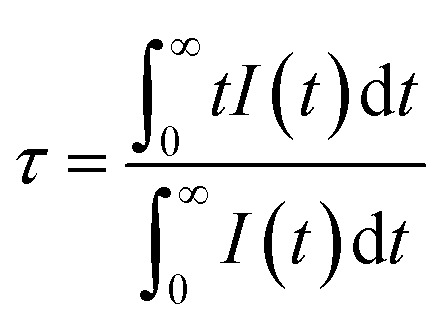
where *I*(*t*) represents the emission intensity at a given time *t* and the integration region covered the entire measured range. The average lifetime values for the ^5^D_3_ and ^5^D_4_ emissions are listed in Table 3. It can be seen clearly that the average lifetime of both bands at 382 nm and 542 nm decreased as the Tb content increased. The lifetime decrease of the ^5^D_3_ emission at 382 nm can be attributed to the occurrence of cross-relaxation energy transfer between Tb^3+^ ions. The lifetime of the ^5^D_4_ emission at 542 nm is not expected to change as a result of cross-relaxation energy transfer although its decay curve can become complex^10,17,29^ due to transitions both from and to this level. The observed lifetime decrease of the emission at 542 nm may be attributed to other concentration quenching mechanisms, possibly due to increased migration of energy and likelihood of encountering quenching sites or an increase in defects affecting the non-radiative transition rate. The cross-relaxation rate (*W*_CR_) can be found by analysing the ^5^D_3_ lifetime values using^30^2
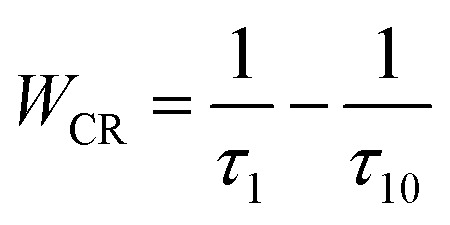
where *τ*_10_ is the lifetime for the band at 382 nm of the sample with the lowest Tb concentration (for which cross-relaxation is assumed to be negligible) and *τ*_1_ is the lifetime of samples with stronger doping. The cross-relaxation efficiency (*η*_CR_) is given by3
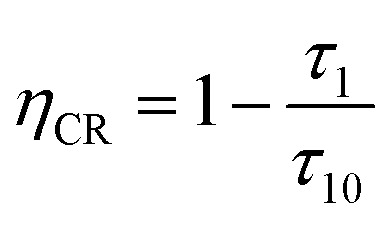


**Table 3:** Average lifetime of the bands at 382 nm and 542 nm for the CaF_2_ doped with different concentration of Tb^3+^ ions

Tb concentration (mol%)	Lifetime (ms) Exc@257 nm		Lifetime (ms) Exc@486 nm	*W*_CR_ (s^−1^)	*η*_CR_ (%)
	382 nm band	542 nm band	542 nm band		
0.25	8.8	22.5	15.6	—	—
0.5	8.5	21.4	14.5	4	3.4
1	8.4	20.9	13.1	5	4.5
2	7.9	20.1	11.3	13	10.2
4	7.8	17.5	10.0	15	11.4
6	7.2	17.4	9.4	25	18.2
8	6.9	14.5	8.3	31	21.6
10	6.6	12.4	7.4	38	25.0

Both the cross-relaxation rate and efficiency, listed in Table 3, increased with Tb^3+^ concentration.

**Fig. 8:**
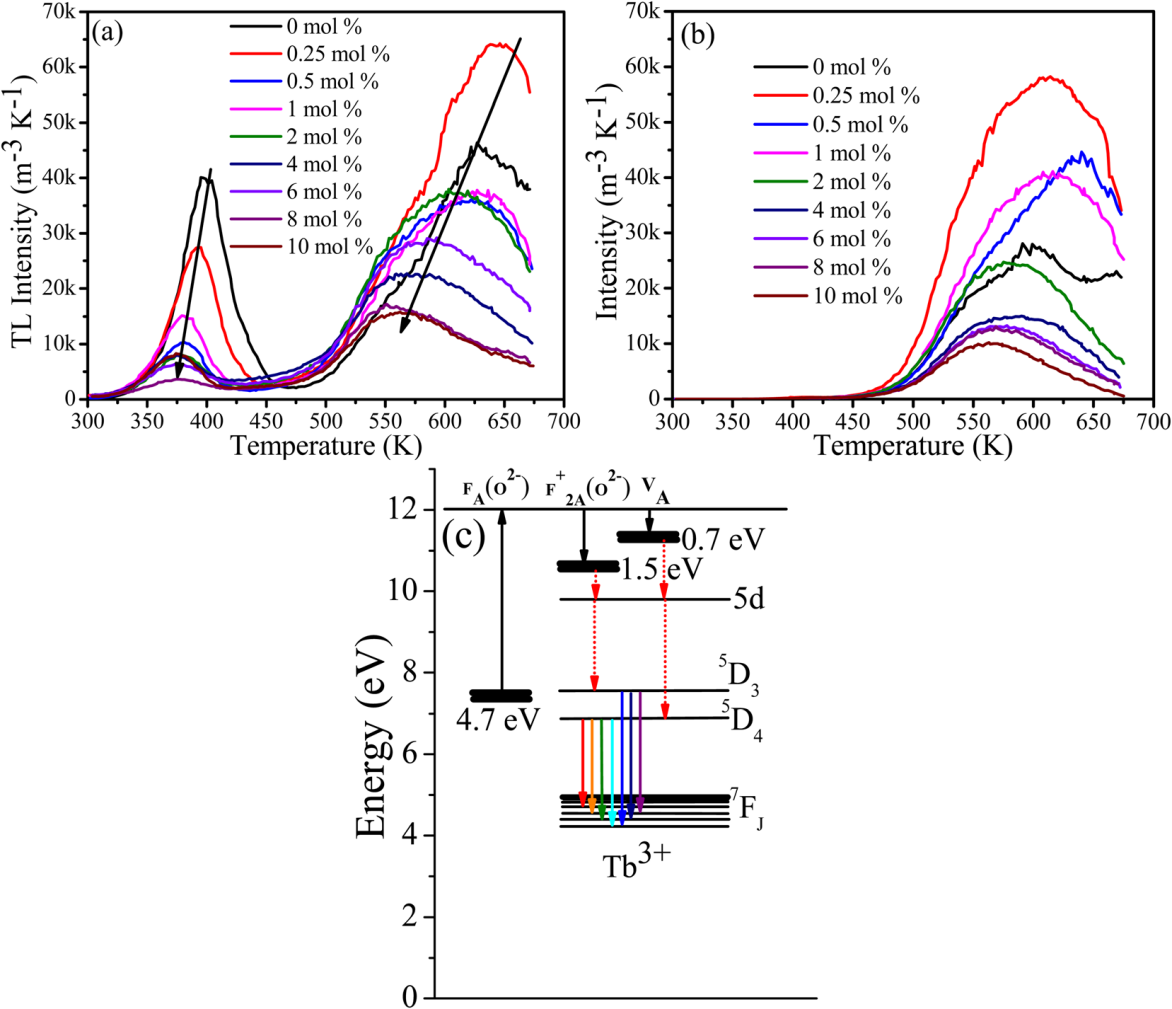
TL glow curve of CaF_2_:Tb^3+^ after 5 min of (a) UV (254 nm) and (b) blue (486 nm) irradiation. (c) Schematic energy level diagram for defects and Tb^3+^ ions.

Generally, the average lifetimes of ^5^D_3_ and ^5^D_4_ emissions of Tb^3+^ ions in samples measured and reported in Table 2 are longer than those usually reported for Tb^3+^ ions.^9,19,31^ Wei Zheng *et al.* observed a long lifetime (12 ms) of Tb^3+^ ion in their CaF_2_:Ce^3+^, Tb^3+^ tri-doped with Na^+^^3^ and such longer lifetimes are expected from Tb^3+^ ions occupying centrosymmetric sites for which forced electric dipole transitions are not allowed and emission occurs due to weak magnetic dipole transitions.^32^ However, the observed lifetimes for the 542 nm band of over 20 ms for lower doping concentrations (Table 3) are extraordinarily long and this might be due to the presence of native traps in CaF_2_ host that were filled during UV (257 nm) excitation source and slowly released during decay measurements, causing the lifetime to increase. To justify this argument, the lifetimes of the band at 542 nm were measured while the samples were excited at 486 nm. The average lifetime of all samples were then shorter than when excited at 257 nm (although similarly decreasing as a function of Tb concentration). This implies that excitation with longer wavelength did not activate the traps which were responsible for extending the lifetimes to the remarkably long value observed when exciting at 257 nm. To further investigate this assumption, TL glow curves of the samples were measured following 257 nm and 486 nm irradiation (Fig. 8).

### 3.5 Thermoluminescence

An equal quantity of CaF_2_:Tb^3+^ powder was rapidly heated to 673 K and cooled down, irradiated with 257 nm (UV) or 486 nm (blue light) for 5 min, and then their TL glow curves were measured. In all samples irradiated at 257 nm two broad bands are present, one at a low temperature of about 400 K due to the shallow traps and the second band at a higher temperature of about 640 K due to deep traps. Generally, the TL glow curve intensity decreased and shifted to a lower temperature with increasing Tb concentration. This indicates that the effective trap density and activation energies were reduced by the addition of the Tb content.^33^ For the samples irradiated at 486 nm, only the TL glow curve due to the deep traps was observed at about 640 K. The TL results support two ideas: (i) the observed long Tb^3+^ lifetime can be ascribed to the presence of traps, and (ii) the reduction in the PL lifetime of Tb^3+^ excited at longer wavelengths could also be associated with unsuccessful activation of traps, although cross-relaxation energy transfer cannot be ignored since it was evidenced by the intensity decreased of ^5^D_3_ emission as a function of Tb content. In previous studies of the cross-relaxation energy transfer of Tb^3+^ ions, the lifetime of ^5^D_3_ energy level was drastically decreased whereas the lifetime of ^5^D_4_ was almost unaffected,^9,12^ unlike in our case where both lifetimes were decreased. Surprising when the samples were irradiated with a longer wavelength of 482 nm (blue light), only the TL glow curve peaks associated with deep traps and not shallow traps were observed. This is difficult to explain in terms of the different TL excitation dynamics, which is not necessarily an energy dependent process.^34^ Another explanation can be deduced from the one-trap-one-recombination model of TL, in which an electron is promoted from the valence band to the conduction band following an irradiation with high energy photon (above the band gap energy). Some of these promoted electrons are trapped instead of returning to the valence band; subsequent thermal energy (heat) will de-trap them to the conduction band from which they decay down to the recombination centre and emit light (TL). In this process the excitation energy required to fill traps is in principle equal to or greater than the energy difference between the valence band and conduction band (bandgap).^35^ Since the excitation wavelength in both cases (257 nm and 486 nm) were less than the band gap, an intermediate sub-band excitation mechanism could be the case. A fluorine centre perturbed by substitutional oxygen [F_A_(O^2−^)] has been reported to form an energy level at 4.7 eV below the conduction band, from which an electron is excited to the conduction band and then trapped at F^+^_2A_(O^2−^) and O^2−^–V_A_ centres at 0.7 eV and 1.5 eV below the conduction band,^14^ respectively (Fig. 8(c)). This may explain the TL glow curve of the samples when irradiated at 257 nm (Fig. 8(a)), where electrons excited from F_A_(O^2−^) centre to the conduction band and trapped at F^+^_2A_(O^2−^) and O^2−^–V_A_ sites, followed by emission of TL glow curve due to shallow and deep traps subsequent to thermal stimulation. However, when the samples were irradiated at 486 nm, which is less energy than the energy required to promote an electron from F_A_(O^2−^) centre to the conduction band, another higher sub-band level may be involved. The exact defect causing this sub-band energy level is yet to be established and hence this needs further investigation. Another possible explanation, according to our observations (Fig. 8(b)), is that an excited electron can reach its trap without being excited to the conduction band *i.e.*, if the excitation energy is equal or greater than the energy difference between the valence/sub band and the trap energy level, trapping may take place; however, during de-trapping *via* thermal energy supply, the trapped electron may have to be elevated to the conduction band in order to find its path to its recombination centre. The assumption may be possible due to the fact that thermal energy increases the phonon's energy that might be involved in elevating the trapped electron into the conduction band during de-trapping process. Kawano *et al.*^36^ studied the dosimetry properties of Tb doped CaF_2_ translucent ceramic under X-ray excitation and found that only the TL glow curve at lower temperature (90–110 °C) due to shallow traps were present. This may support our explanation that higher photon energy tends to fill the shallow traps where lower photon energy fill deep traps. Their PL decay curve lifetime excited at 340 nm was only several milliseconds, which is much lower than the average lifetimes obtained in this study. This is again contradicting our assumption that the shallow traps were responsible for such longer lifetimes. These differences may be attributed to the differences between the sample's nature. Godbole *et al.* observed a drastic drop in the TL intensity of CaF_2_:Tb phosphor after sequential irradiation with 250 nm followed by 365 nm, and they suggested that irradiation with longer wavelength facilitated de-trapping.^34^

### 3.6 Temperature-dependent photoluminescence

**Fig. 9:**
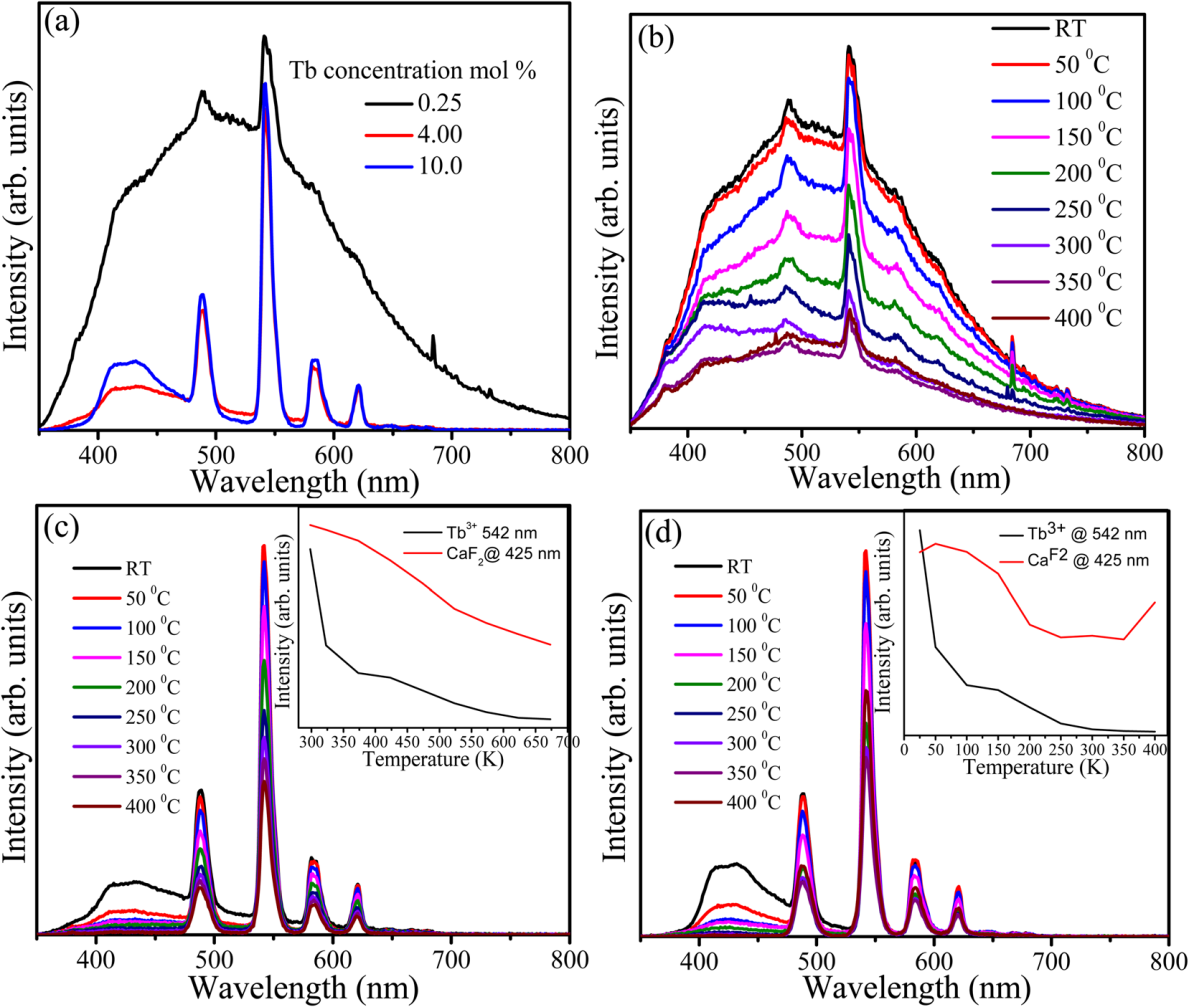
PL spectra of the CaF_2_ doped with different Tb concentrations (a), and temperature-dependent PL of the same samples (b) 0.25 mol%, (c) 4 mol% and (d) 10 mol%, the insets are the PL integrated intensity of the 542 nm band and the broad band ranges from 400 nm to 476 nm. All samples were excited at 325 nm by using He–Cd laser.

CaF_2_ always contains native defects such as vacancies and interstitials of Ca^++/+^ and F^−^. These defects form energy levels within the band gap and act as luminescence centres and traps. In addition to the native defects, impurities (particularly oxygen ions) are also presents in the CaF_2_ matrix and interact with the native defects to form complexes. Generally, the presence of various types of defects in the CaF_2_ matrix gives rise to PL in the violet and visible regions as well as the TL glow curve.^15,32^ PL spectra excited at 325 nm with a He–Cd laser were recorded at room temperature for samples containing 0.25, 4 and 10 mol% of Tb^3+^ ions (Fig. 9(a)). The PL spectrum of the sample doped with 0.25 mol% possesses two types of emissions: a very broad band extending from violet to the visible region which is attributed to the presence of defects and their complexes in the CaF_2_ matrix, and the characteristic narrow band emission of Tb^3+^ ions overlaying the broad emission. Upon increasing the Tb concentration, the broad band drastically decreased (especially the part of the spectrum above 500 nm) whereas the emission below 500 nm was still present at a relatively higher intensity. The broad band emission may be divided into two parts, below and above 500 nm, and they are therefore attributed to different types of defects. Two reasons for the reduction in the broad emission band upon increasing the Tb concentration were considered. First energy transfer occurs from these defects to the Tb^3+^ ions, although this is unlikely because there is no increase in the characteristic PL intensity of the Tb^3+^ ions. The second possibility is the elimination and migration of the native defects of CaF_2_ due to the increase in Tb content. The XRD results predicted the expansion of the lattice parameters and creation of interstitial F^−^ ions as the Tb concentration increased. Since the F content was fixed during the hydrothermal reaction, F^−^ ions may have migrated from an active defect site where they contributed to the defect emission into an interstitial (inactive) site where they did not contribute to the defect emission. Elimination of other type of defects facilitated by the migration and formation of F^−^ interstitial is also possible. Nevertheless, the precise dynamics of CaF_2_ native defects is very complex and is yet to be fully resolved. The samples with different doping were measured at different temperatures (Fig. 9(b–d)) and in all cases the PL intensity exhibited a decrease when the temperature increased. This may be understood by the temperature-induced phonon energy mechanism which created more nonradiative channels and hence the PL intensity decreased. As shown in the insets of Fig. 9(c and d), the PL intensity of the Tb^3+^ ions and the CaF_2_ defects were decreased as a function of temperature. The defect emission intensity was rapidly decreased at 325 K and then kept decreasing with lower rate until it diminished at 600 K, whereas the Tb^3+^ intensity decreased with lower rate and still maintained 60% of its intensity at 675 K. It is well known that 4f–4f transitions of lanthanide ions are protected by the outer orbitals and therefore not very sensitive to external perturbations. For the sample containing 10 mol% of Tb^3+^ ions (Fig. 9(d)), the defect emission intensity showed similar behaviour to the sample with 4 mol% of Tb^3+^ ions (Fig. 9(c)). However, the Tb^3+^ emission intensity demonstrated different behaviour than its counterpart with lower Tb concentration. Its emission intensity initially increased at 323 K and then decreased until 480 K, after which it was stabilized and slightly increased at the maximum temperature (675 K). Generally, the sample containing 10 mol% of Tb maintained about 60% of its Tb^3+^ emission intensity at high temperature (above 480 K). This may be ascribed to presence of low defect concentration.

### 3.7 Stability studies

**Fig. 10:**
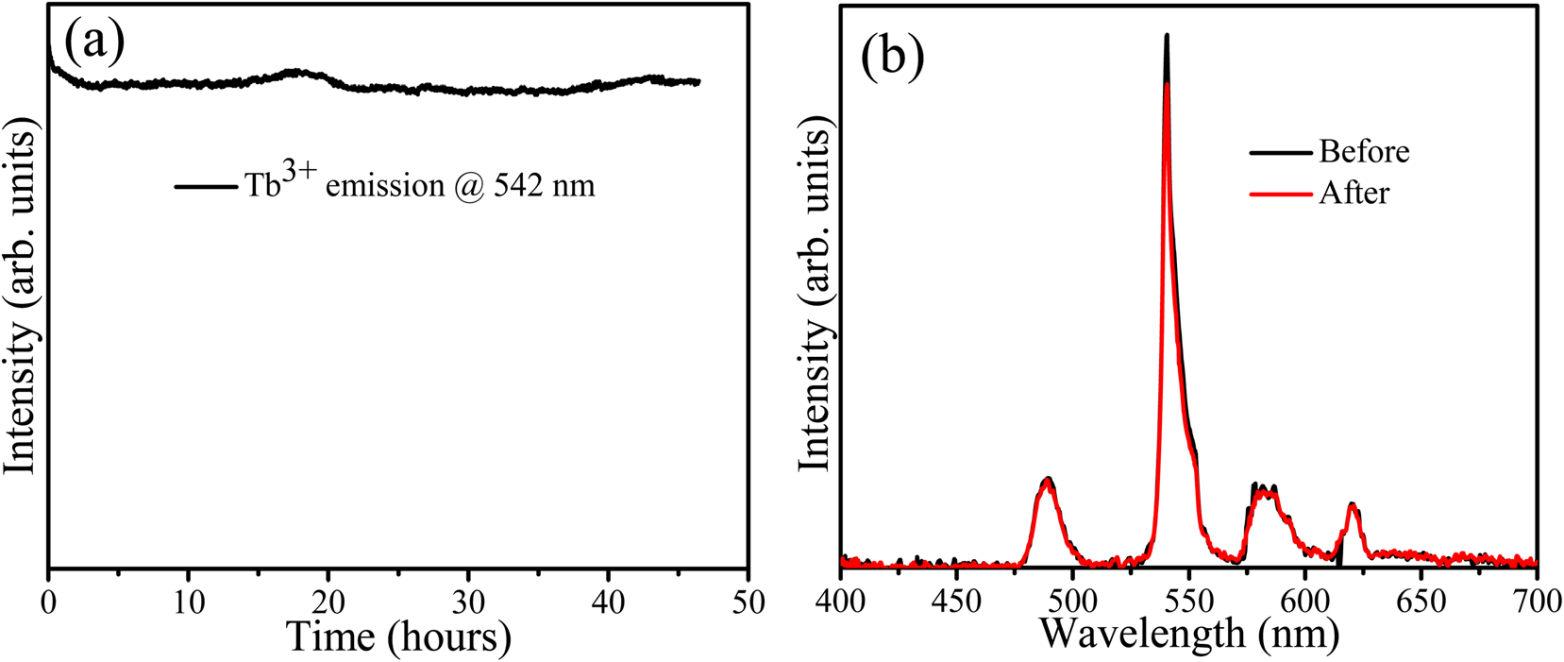
(a) PL stability, (b) PL spectra before and after irradiation.

The sample containing 10 mol% Tb^3+^ ions was irradiated with 254 nm UV light for about 46 h to assess its stability. The PL spectra were recorded every minute and the PL intensity variation of the Tb band at 542 nm is depicted in Fig. 10(a) as a function of time. It decreased slightly during the first 4 h and then stabilized. This could be attributed to some surface modification due to the adsorption of atmospheric oxygen-related species during irradiation as indicated by the O 1s XPS results (Fig. 4). Godbole *et al.* irradiated CaF_2_:Tb^3+^ by sustained 258 nm UV light for 5 min and recorded its PL intensity at 546 nm, but found no significant variation in the PL intensity during this short period of irradiation.^37^ In 2011 Mamykin *et al.* reported the degradation of UV irradiated CaF_2_:Tb together with the appearance of a brown colour which was associated with the conversion of Tb(iii) to Tb(iv) ions.^38^ However, our XPS results (Table 2) comparing the irradiated and unirradiated samples indicated no change in the ratio of Tb ions in the two valence states. The shorter wavelength of 215 nm used during this previous study may have contributed to these different findings. The PL spectra measured in this study before and after 47 h of irradiation are shown in Fig. 10(b) and no notable changes in the profile can be observed.

**Fig. 11:**
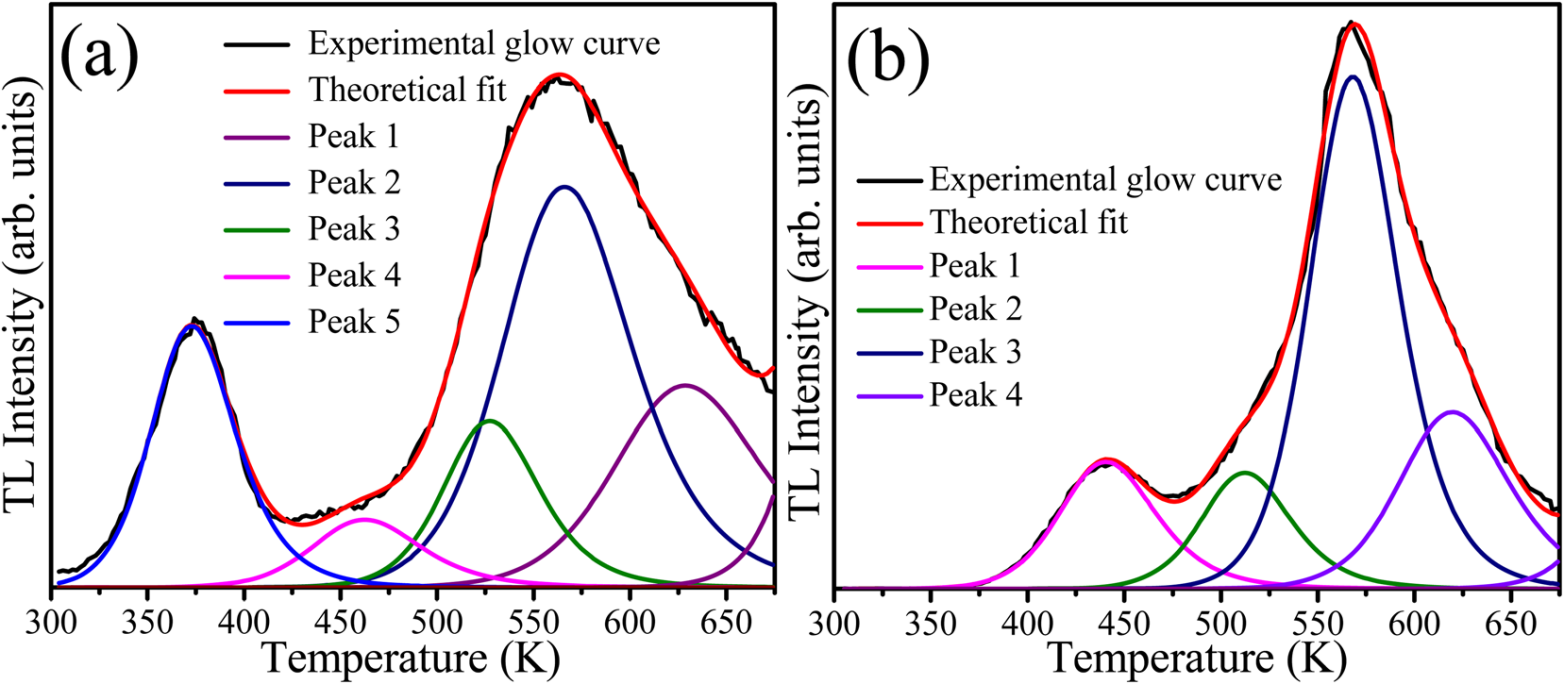
Deconvoluted TL glow curves of the sample with 10 mol% of Tb ions (a) unirradiated and (b) irradiated with 254 nm UV light for 46 hours.

The presence of defects in the CaF_2_ crystal plays a crucial role in PL dynamics. These defects include chemical impurities, vacancies, and interstitials. In addition the chemical impurities, these defects can be classified into two categories: positively charged defects such as Ca^2+^-related defects, and or negatively charged defects such as F^−^. Positively charged defects act as electron traps whereas the oppositely charged defects act as traps for holes.^39^ TL measurements provide a practical method for studying the defects/traps in such materials. Therefore, the TL glow curves were measured for the sample with 10 mol% Tb^3+^ ions before and after irradiation with 254 nm UV light for 46 h as shown in Fig. 11. The unirradiated sample exhibited a broad TL glow curve consisting of five bands. Two bands in the lower temperature region at about 380 K and 460 K were facilitated by shallow traps, and the other three bands in the higher temperature region at about 525, 572, and 625 K were ascribed to deep traps. The irradiated sample exhibited only four bands, and the TL band was absent at the lowest temperature. This indicates that UV irradiation eliminated some of the defects responsible for the shallow traps, which might explain the slight decrease in the PL intensity during irradiation. The TL glow curves provide information about the traps/defect energies, order of kinetics, density, and escaping/frequency factors. To obtain these parameters, the glow curve was deconvoluted using the first, second or general order kinetic equations. The first order kinetic equation assume that the trapped electrons were recombined with their hole pairs after thermal energy supply, whereas the second order kinetic considers that re-trapping has taken place. When deconvolution using the first order kinetics is not possible, the second order kinetic equation are used, otherwise the general order kinetic equation is used for deconvolution. In our case, deconvolution using the first order kinetic equation did not match well with experimental TL glow curve, and hence the second order kinetic equation^40^4
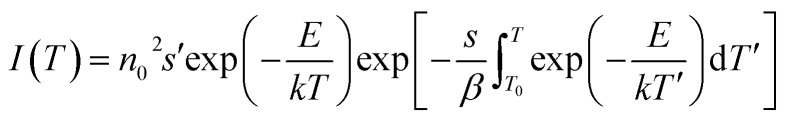
was used, where *n*_0_ is the trap density, *s* is the escape or frequency factor, *β* represents the heating rate, *E* the trap depth, *k* is Boltzmann's constant, and *T* is the absolute temperature. TLAnal software^41^ was used for the deconvolution and the TL parameters value are listed in Table 4. A figure of merit of less than 5% may be considered reasonable for fitting TL glow curves, and values of 2.5% and 2.7% for fitting the TL glow curves of the samples before and after UV irradiation were obtained, respectively. The elimination of some native defect of CaF_2_ during irradiation, as evidenced by the disappearance of the lowest temperature TL peak, may also have been responsible for the slight initial degradation during UV irradiation. Moreover, changes in the TL kinetics of the deconvoluted peaks after UV irradiation indicate changes in the local chemical environment in the vicinity of other defects induced as a result of UV irradiation.

**Table 4:** Trapping parameters of the unirradiated and 254 nm UV-irradiated sample with 10 mol% of Tb ions

Sample	Peak	Activation energy (eV)	Frequency rate (s^−1^)	Trap density (cm^−3^)
Unirradiated	1	0.758	5.485 × 10^4^	9.472 × 10^4^
	2	0.905	5.239 × 10^4^	3.162 × 10^4^
	3	1.359	3.849 × 10^7^	6.828 × 10^4^
	4	1.131	9.997 × 10^3^	2.243 × 10^5^
	5	1.227	8.900 × 10^3^	1.283 × 10^5^
Irradiated	1	0.936	7.599 × 10^5^	1.740 × 10^5^
	2	1.382	7.688 × 10^7^	1.477 × 10^5^
	3	1.715	7.340 × 10^8^	6.481 × 10^5^
	4	1.630	1.514 × 10^7^	2.780 × 10^5^

## 4 Conclusions

Tb^3+^ doped CaF_2_ nanoparticles were successfully synthesized by a hydrothermal method. The luminescence due to the Tb^3+^ ions and native point defects of CaF_2_ were investigated. The stability of the phosphor under continuous UV irradiation and high temperatures was also studied. Two findings can be inferred as the Tb content increased: (i) relocation of F^−^ ions into interstitial sites accompanied by the elimination of other types of defects and reduction of traps observed by TL measurements, and (ii) cross-relaxation energy transfer between Tb^3+^ ions was evident from the PL and lifetime measurements. The sample with the highest Tb doping (10 mol%) retained approximately 60% of its ^5^D_4_ PL intensity at 473 K and above, and it degraded only marginally under prolonged UV irradiation for 46 h, although TL analysis indicated the elimination of some shallow traps as a result of prolonged illumination with 254 nm UV light. These findings indicate that the native point defects of CaF_2_ play an important role in the luminescence dynamics of Tb^3+^ ions and must be taken into consideration in further studies.

## Author contributions

E. H. H. Hasabeldaim: conceptualization, formal analysis, investigation, methodology, write – original draft. H. C. Swart: supervision, writing – review, funding acquisition and editing of paper. R. E. Kroon: conceptualization, project administration, methodology, resources, supervision, writing – review & editing of paper.

## Conflicts of interest

There are no conflicts to declare.

## Supplementary Material

RA-013-D2RA07897J-s001
