# Supplementary material for: Luminescence and stability of Tb doped CaF2 nanoparticles
Source: RSC Adv. 2023 Feb 13;13(8):5353–66. doi: 10.1039/d2ra07897j (PMC9924223; doi:10.1039/d2ra07897j)
Supplement: RA-013-D2RA07897J-s001 [file RA-013-D2RA07897J-s001.pdf]

Electronic Supplementary Material (ESI) for RSC Advances.
This journal is © The Royal Society of Chemistry 2023

Luminescence and stability of Tb doped CaF_2 nanoparticles

E. H. H. Hasabeldaim, H. C. Swart and R. E. Kroon**

Department of Physics, University of the Free State, Bloemfontein, 9300, South Africa

Department of Physics, University of the Free State, PO Box 339, Bloemfontein, 9300, South Africa.

*E-mail addresses: omda180@gmail.com (E. H. H. Hasabeldaim) and KroonRE@ufs.ac.za (R. E. Kroon).

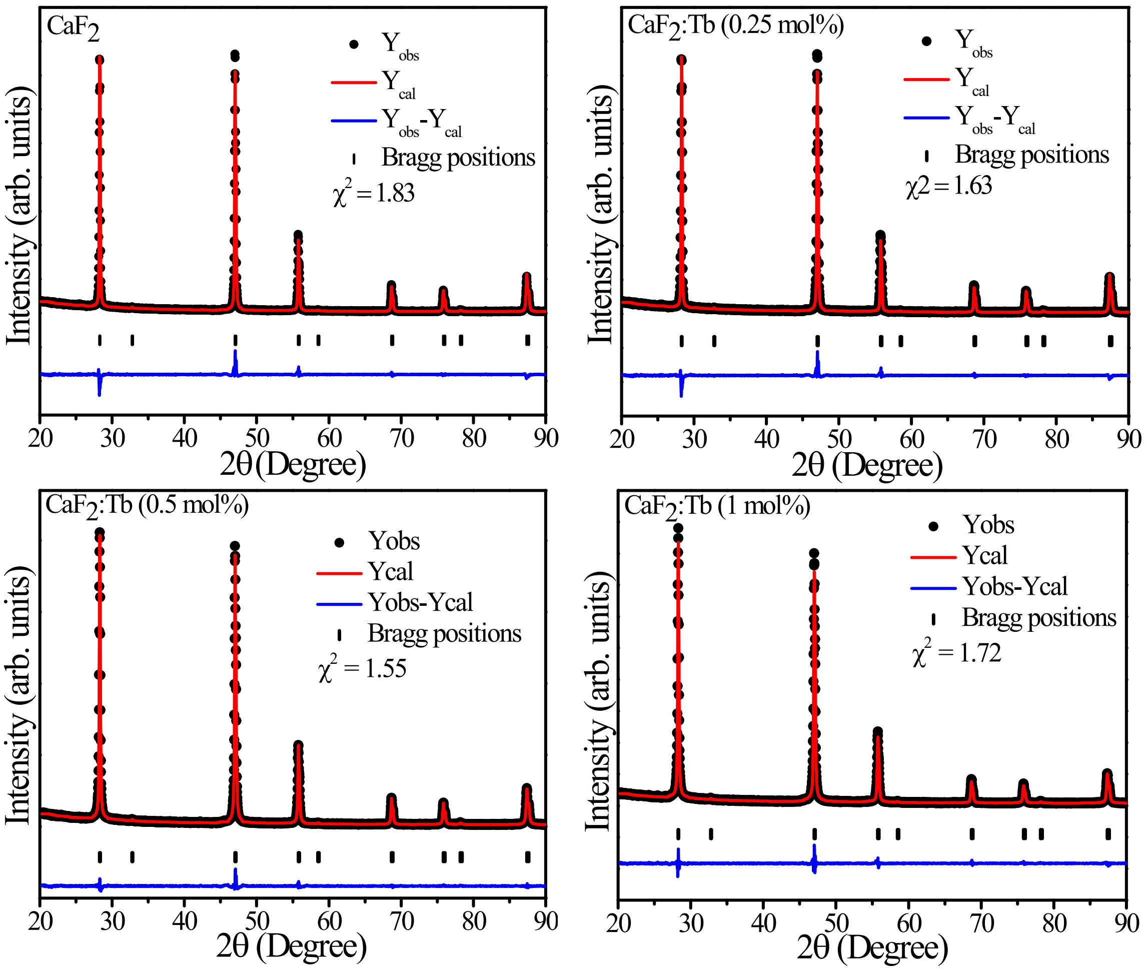

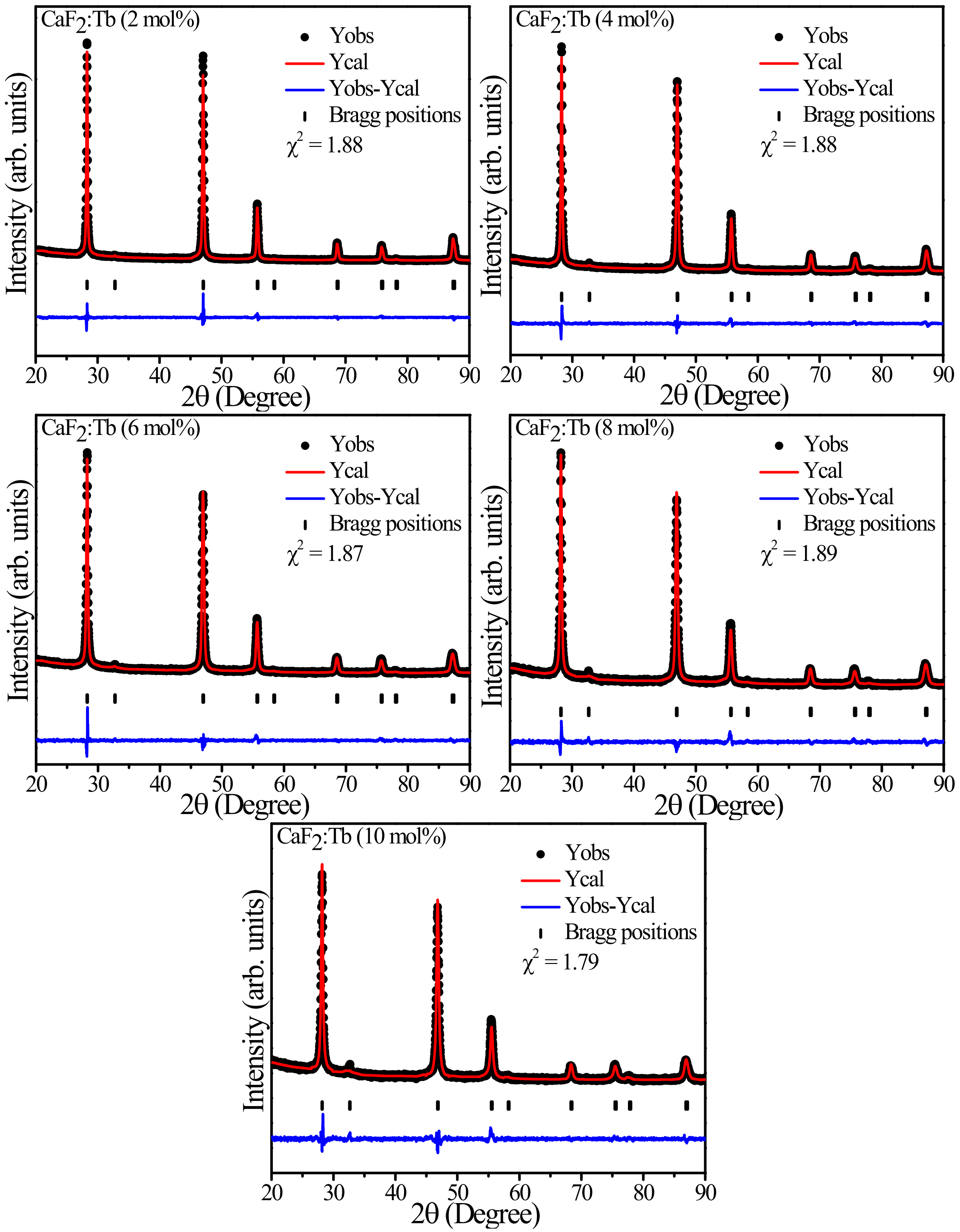

Figure S1. Rietveld refinement of the XRD patterns of CaF_2 nanoparticles doped with different Tb concentrations.

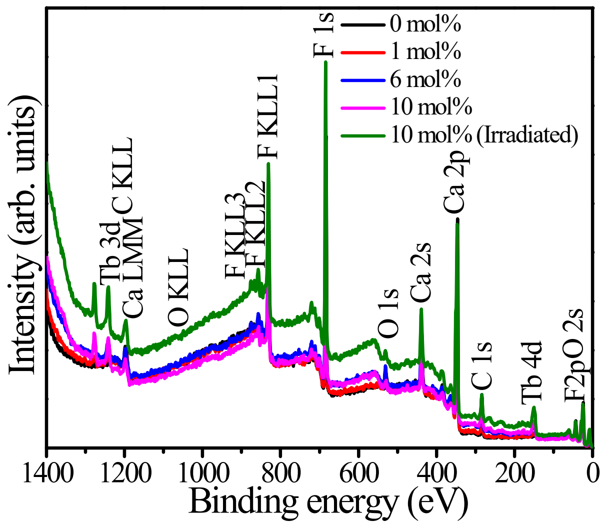

Figure S2. XPS wide survey spectra of CaF_2 nanoparticles doped with different Tb concentrations.

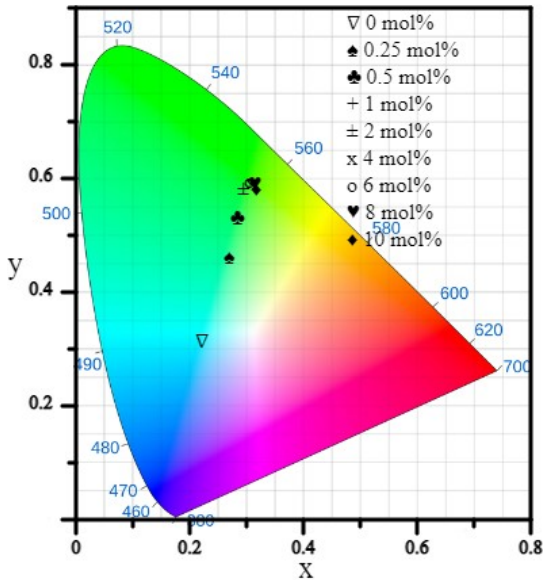

Figure S3. CIE chromaticity diagram of the CaF_2 samples with different Tb content.

Table S1. CIE coordinates (X and Y) and colour correlated temperature (CCT) of the CaF_2 samples containing different Tb concentration.

Sample	X	Y	CCT (K)
0 mol%	0.22	0.31	14446
0.25 mol%	0.27	0.46	7235
0.5 mol%	0.28	0.53	6584
1 mol%	0.30	0.58	6194

2 mol%	0.30	0.58	6194
4 mol%	0.31	0.59	5908
6 mol%	0.31	0.60	5960
8 mol%	0.31	0.59	5827
10 mol%	0.32	0.58	5757
